# Remodeling technique using T-stenting and coils to treat complex renal aneurysm

**DOI:** 10.1590/1677-5449.200141

**Published:** 2021-06-16

**Authors:** Patrick Bastos Metzger, Kamilla Rosales Costa, Simone Lessa Metzger

**Affiliations:** 1 Escola Bahiana de Medicina e Saúde Pública – EBMSP, Salvador, BA, Brasil.; 2 Obra Sociais Irmã Dulce – OSID, Hospital Santo Antônio, Salvador, BA, Brasil.

**Keywords:** aneurysm, renal artery, endovascular procedures

## Abstract

Renal artery aneurysm is a rare condition that is being diagnosed with increasing frequency because of wider use of angiotomography. We describe a case of complex type II renal artery aneurysm in a patient with systemic arterial hypertension and non-dialysis chronic kidney disease. The treatment performed was endovascular repair using the remodeling technique with T-stenting and coils to preserve the renal arterial branches, obtaining satisfactory arteriographic results and good clinical outcomes.

## INTRODUCTION

Renal artery aneurysm (RAA) is a rare condition, with prevalence ranging from less than 0.3% to 1% of the population. The number of cases diagnosed has been increasing because of wider use of imaging methods such as Doppler ultrasonography and computed tomography angiography (CTA).^1^^,^^2^

Indications for treatment and choice of technique are still controversial because of the small number of studies available, which is a consequence of the rarity of the disease, making it difficult to conduct randomized clinical trials.^2^^,^^3^ Open surgery is one treatment option, with excision of the aneurysm and reconstruction or nephrectomy.^2^ As endovascular techniques evolved, treatment of RAA using percutaneous repair became possible, offering reduced surgical trauma, shorter postoperative hospital stay, and lower morbidity, when compared to open surgical treatment.^1^^,^^4^ However, treatment of complex RAA in which it is necessary to preserve branches emerging from the aneurysm that supply the poles of the kidneys remains a therapeutic challenge.^1^^-^4 New techniques and materials developed within interventional neurology appear to offer promising options for treatment of these complex RAA.

We describe endovascular treatment of a complex type II RAA involving the bifurcation of the principal renal artery, with a major branch emerging from the aneurysm to supply the mid pole, using a combination of the remodeling technique with T stents and coiling. The objective was to preserve the arterial branches supplying each of the renal poles in a patient with non-dialysis chronic kidney disease (CKD) and refractory systemic arterial hypertension (SAH). The patient gave her consent to publication of the clinical case and images. The study was approved by the local Ethics Committee (ruling number 4.451.756).

## CASE REPORT

**Figure 1:**
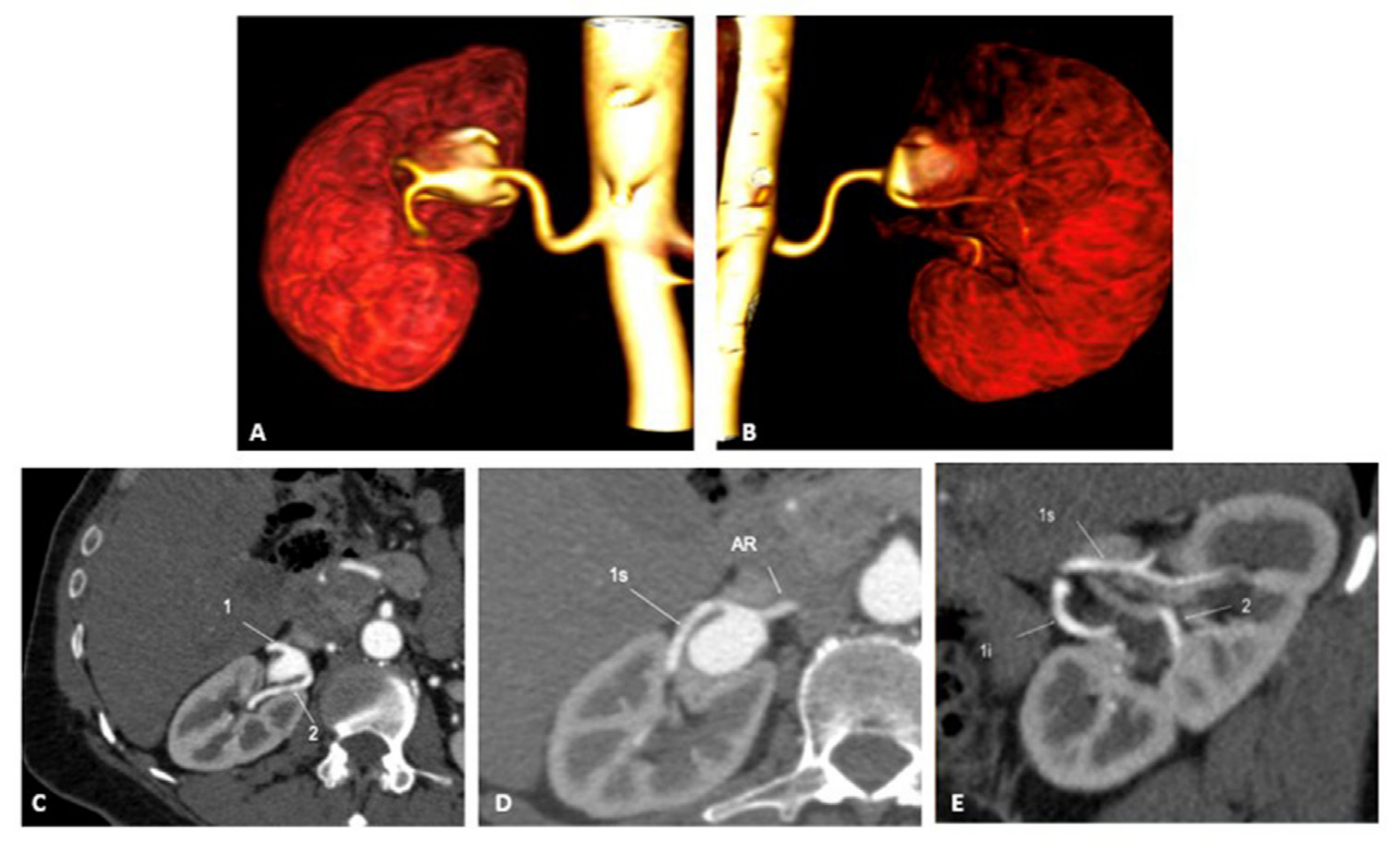
(A) Angiotomography with volumetric reconstruction of the type II renal aneurysm, in anteroposterior view, showing the type II renal aneurysm involving the renal bifurcation; (B) Angiotomography with volumetric reconstruction of the type II renal aneurysm, posteroanterior view, showing the type II renal aneurysm with the branch feeding the mid pole of the kidney emerging from the type II renal aneurysm; (C) Multiplanar angiotomography slice showing in 1 – main renal artery close to the superior and inferior bifurcation; and in 2 – the artery feeding the mid pole emerging from the aneurysm; (D) Multiplanar angiotomography slice showing, at 1s – the branch feeding the upper renal pole; and AR – main renal artery; (E) Multiplanar angiotomography slice showing at 1s – the branch feeding the upper renal pole; 1i – the branch feeding the lower renal pole; and 2 – the branch feeding the mid renal pole.

The patient was a 55-year-old woman with hypertension, dyslipidemia, and stage III CKD who was being monitored by the cardiology team because of hard to control SAH, for which she was taking four antihypertensive drugs. During an examination of the renal arteries with Doppler ultrasound, a large aneurysm was detected in the hilum of the right kidney. Abdominal CTA showed that it was a type II RAA measuring 3.4 x 2.8 cm and involving the bifurcation of the main renal artery, which supplied the superior and inferior poles and a large branch artery that fed the mid pole, originating after the aneurysm (Figure 1). The patient’s creatinine clearance was 41 mL/min/1.73 m^2^.

**Figure 2:**
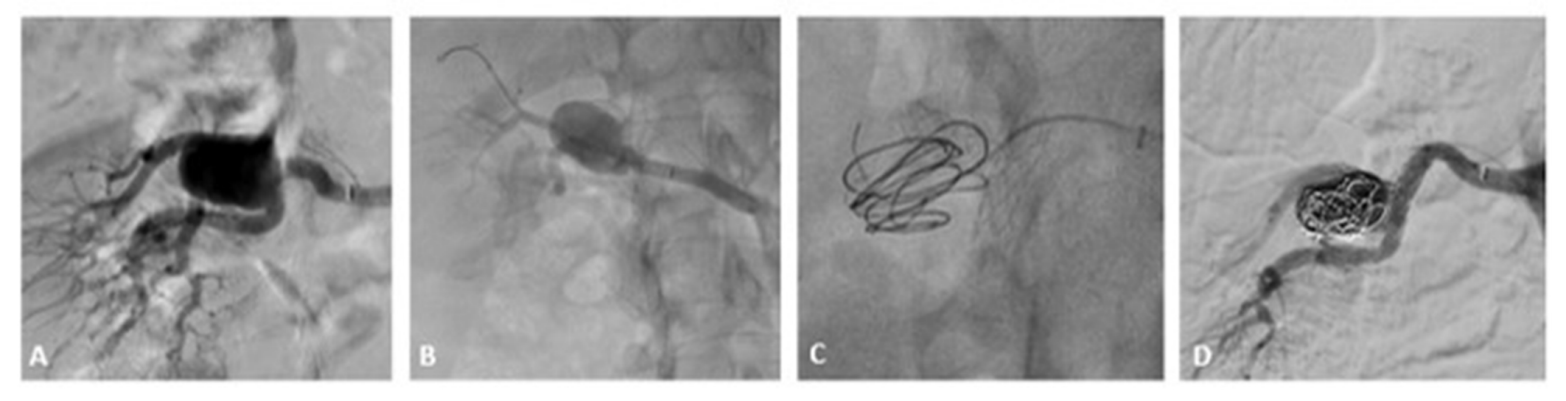
(A) Renal arteriography showing the complex type II renal aneurysm – before treatment; (B) Positioning the first stent at the bifurcation of the main renal artery; (C) Positioning the T stents and starting embolization via the meshes of the stent; (D) Final control arteriography showing patent feeder arteries and embolization of the renal aneurysm.

After careful angiotomographic renal investigation and studying the treatment options that would preserve the greatest proportion of the parenchyma, the decision was taken to conduct endovascular treatment with the remodeling technique using T-stenting and coils to preserve the arteries feeding the kidney. The treatment was initiated using a 6 Fr Destination long introducer sheath (Terumo Medical, Somerset, United States), followed by catheterization of the right renal artery with a cobra 2 catheter and 0.035 x 260 cm hydrophilic guidewire (Terumo Medical, Somerset, United States). After renal arteriography to provide a better view of the bifurcation of the main renal artery (Figure 2A), the upper branch of the main renal artery was catheterized with a 0.035 x 260 amplatz guidewire (Boston Scientific, Minneapolis, United States), followed by deployment of a Palmaz Genesis Pro 5 x 39 mm stent (Cordis Corporation, Warren, United States) (Figure 2B) flush to the bifurcation. The mesh of the Palmaz Genesis Pro stent (Cordis Corporation, Warren, United States) was then catheterized with a Pt2 0.014 x 180 cm moderate support guidewire (Boston Scientific, Minneapolis, United States) and the branch feeding the mid pole was catheterized and stented with a Palmaz Blue 3 x 18 mm stent (Cordis Corporation, Warren, United States), through the mesh of the Genesis Pro stent into the artery feeding the mid pole. Next, a Progreat 2.4F microcatheter (Terumo Medical, Somerset, United States) was advanced through the meshes of the stent and 6 AZUR-18 x 12mm x 20 cm detachable hydrocoil embolization coils (Terumo Medical, Somerset, United States) were deployed into the aneurysm sac (Figure 2C). Control arteriography showed that the branches feeding the kidney poles had been salvaged and that the renal aneurysm sac was embolized (Figure 2D).

**Figure 3:**
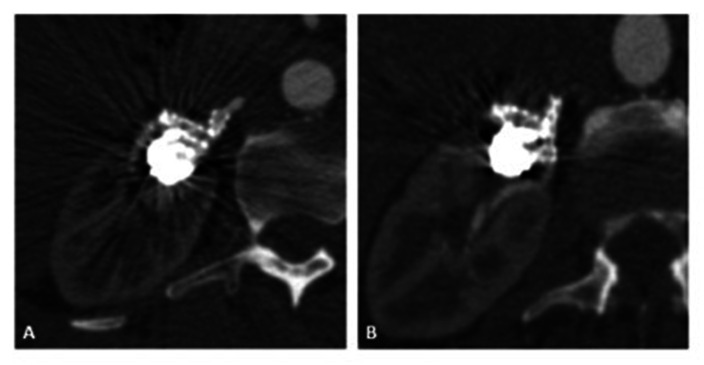
(A) Angiotomographic control at 3 months showing the patent branch feeding the mid renal pole; (B) Angiotomographic control at 3 months showing the patent branches feeding the upper and lower renal poles.

The patient had a satisfactory postoperative course and she was discharged on the third day, with no elevation in nitrogenous wastes or hematuria, on dual antiplatelet therapy. Outpatients follow-up CTA 3 months after the procedure showed the renal parenchyma free from ischemia and all feeder vessels preserved (Figure 3A and 3B). The patient has been in follow-up for 3 years, with good clinical, laboratory, and imaging outcomes. During this follow-up period there has been no need for renal replacement treatment and the patient is still taking four antihypertensive drugs, achieving adequate control of her blood pressure.

## DISCUSSION

Aneurysms of the visceral arteries (VAA) are rare, present in less than 1% of the total population. Renal artery aneurysms are one of the less frequent types of VAA, accounting for around 15 to 22%.^4^ Autopsy studies indicate a 0.1% incidence of RAA. However, studies undertaken using renal arteriography found evidence that incidence varies from 0.3 to 1%.^1^^,^^2^^,^^5^ This disease is more common in females, because of the strong association with renal fibromuscular dysplasia,^1^^,^^6^ but it is also linked to atherosclerosis, infections, trauma, Kawasaki disease, Marfan Syndrome, vascular dysplasias, and polyarteritis nodosa (PAN).^1^^,^^3^ The majority of patients are asymptomatic, but some present with symptoms such as SAH, renal ischemia, hematuria, and flank pain, but the relationships are not well established. Complications involve renal vascular hypertension, renal thrombosis and emboli, renal infarct, and rupture.^7^ Although rupture is a relatively infrequent phenomenon (5 to 10%), it is associated with high mortality rates (80%).^1^ In our case, the RAA was an incidental finding during investigation of refractory SAH of renovascular etiology.

Studies such as those by Barros et al.^1^ and Abath et al.^7^ demonstrate that type II RAA are more common than other types. This type of aneurysm is defined as challenging in the literature, particularly when adjacent to arterial bifurcations, and open surgical treatment (endoaneurysmorrhaphy, nephrectomy, or autotransplant) has been the established treatment for decades.^6^^,^^7^ More recent studies recommend endovascular repair using remodeling techniques with stenting in conjunction with coils or liquid embolization agents, with the objective of salvaging the native renal vessels when the aneurysm is located in the main renal artery or involves bifurcations in cases of saccular RAA with wide necks.^1^^,^^2^^,^^7^ Even so, these lesions are a challenge to treat with this type of repair because of the difficult anatomy, demanding more complex techniques.^1^^,^^7^ Along the same lines, fusiform type II RAA are still very difficult to treat percutaneously.^1^^,^^7^ Notwithstanding, the efficacy of endovascular repair has been confirmed at 89.7 to 98% of cases, with reduced morbidity, operating time, and length of postoperative hospital stay, in addition to reduced surgical trauma.^1^^,^^4^^,^^7^

The indications for RAA treatment are still uncertain in the literature, but there is consensus that treatment is indicated for symptomatic patients, large aneurysms, cases with renal embolization, aneurysm in pregnant women or women of fertile age, and aneurysms > 2.5 cm.^1^^,^^2^^,^^6^^-^12

Until techniques for remodeling with stents were developed, saccular aneurysms with wide necks (sac:neck ratio < 2) could not be treated by selective embolization because of the significant risk of occlusion of the main vessel caused by migration of the coils or liquid embolization agent into the unprotected artery. With this technique, a stent is deployed into the main artery, at the neck of the aneurysm, while microcoils or other embolization agents are deposited into the aneurysm through the mesh of the stent, which provides support, avoiding inadvertent migration of the emboligenic materials, in addition to contributing to changing the local hemodynamic parameters, diverting flow and providing a scaffold for endothelization at the site, reducing the likelihood of recanalization of the aneurysms over the long term.^1^^,^^2^^,^^7^^-^12 Saccular aneurysms with narrow necks (sac:neck ratio > 2) are candidates for primary embolization of the aneurysm sac with coils or liquid embolization agents.^1^^,^^9^^-^12

Use of stents primarily employed in interventional neurology, such as the Solitaire (Medtronic, Minneapolis, United States), provides great navigability and flexibility, since they can pass through microcatheters. These stents also offer the advantage that they can be repositioned even after they have been fully released. However, more studies reporting long-term results of use of these stents in RAA are needed.^10^ In our case, these stents were not available for use.

Recent technological developments involve endovascular techniques using flow-modulating stents. These stents have multiple layers specifically designed to reduce the velocity of blood flow into the aneurysm sac, provoking thrombosis while maintaining flow through the main artery and its branches. They are widely used in interventional neurology, but their use in peripheral vessels still lacks evidence, with only small case series and reports.^9^^,^^10^

In the case described, we observed a large artery feeding the mid pole with origin in the type II RAA in a patient with stage II CKD, in whom it was necessary to save the renal parenchyma. After detailed analysis of several CTA reconstructions and calculations of the arteries upstream and downstream of the aneurysm, we chose to conduct treatment that would conserve the renal artery, employing Nitinol balloon-expandable stents and using the meshes of the stent as support to salvage the branch feeding the mid pole and as support for the coils, preventing them from migrating. After deployment of the stents, we filled the aneurysm sac with hydrocoil embolization coils in order to embolize the aneurysm sac. Intraoperative control arteriography and control angiotomography at 3 months both demonstrated patency of the branches treated, absence of renal ischemia, and embolization of the aneurysm sac.

The combination of techniques that were performed for this patient is complex to execute, requiring a rigorous angiotomographic study beforehand in order to assess the anatomy and adequately plan the procedure. Thus, vascular anatomy permitting, it is possible to repair complex type II RAA while saving the native vessels in the renal territory, as was achieved in this case. There is no doubt that borderline renal function combined with a complex RAA imposes the need to preserve the kidney and this remodeling technique using T-stenting and coils is well-indicated in these cases. Nowadays, knowledge about new materials and techniques derived from interventional neurology has made treatment of complex RAA feasible and effective, which should influence treatment choices.
