# Supplementary material for: Remodeling technique using T-stenting and coils to treat complex renal aneurysm
Source: J Vasc Bras. 2021 Jun 16;20:e20200141. doi: 10.1590/1677-5449.200141 (PMC8218831; doi:10.1590/1677-5449.200141)
Supplement: Translation

# Translation: Técnica de remodelamento com uso de stents em T e molas para tratamento de aneurisma renal complexo

**Authors:** Patrick Bastos Metzger, Kamilla Rosales Costa, Simone Lessa Metzger

**Affiliations:** 1 Escola Bahiana de Medicina e Saúde Pública – EBMSP, Salvador, BA, Brasil.; 2 Obra Sociais Irmã Dulce – OSID, Hospital Santo Antônio, Salvador, BA, Brasil.

**Keywords:** aneurisma, artéria renal, procedimentos endovasculares

## Abstract

O aneurisma da artéria renal é uma condição rara, que vem sendo cada vez mais diagnosticada devido ao uso mais amplo da angiotomografia. Descrevemos um caso de aneurisma da artéria renal tipo II complexo em uma paciente com hipertensão arterial sistêmica e doença renal crônica não dialítica. O tratamento estabelecido foi o reparo endovascular através da combinação da técnica de remodelamento com stents em T e molas, para a preservação dos ramos arteriais renais. Foram obtidos resultados arteriográficos satisfatórios e boa evolução clínica.

## INTRODUÇÃO

O aneurisma de artéria renal (AAR) constitue uma afecção incomum, tendo uma prevalência de menos de 0,3 a 1% da população. Atualmente, devido ao maior uso de métodos de imagem como a ultrassonografia com Doppler e a angiotomografia computadorizada (ATC), o número de diagnósticos vem aumentando^1^^,^^2^.

As indicações para o tratamento e a escolha da técnica ainda são controversas devido ao número reduzido de estudos disponíveis, por se tratar de uma doença rara, o que dificulta a realização de ensaios clínicos randomizados^2^^,^^3^. A cirurgia aberta pode ser realizada através da excisão do aneurisma com reconstrução ou nefrectomia^2^. Com o avanço da técnica endovascular, o tratamento do AAR através do reparo percutâneo tornou-se possível, proporcionando uma redução do trauma cirúrgico e do tempo de internação pós-operatória, bem como da morbidade, quando comparada ao tratamento cirúrgico aberto^1^^,^^4^. No entanto, o tratamento dos AAR complexos, em que a preservação dos ramos para a irrigação dos polos renais emergem do aneurisma, continua sendo um desafio terapêutico^1^^-^4. Novas técnicas e materiais derivados da neurointervenção parecem alternativas promissoras para o tratamento desses AAR complexos.

Relatamos o tratamento endovascular de um AAR tipo II complexo, envolvendo a bifurcação da artéria renal principal, com saída de um grande ramo polar para o polo médio do aneurisma através da combinação da técnica de remodelamento com stents em T e molas. O objetivo foi preservar os ramos arteriais de cada polo renal em uma paciente com doença renal crônica (DRC) não dialítica e hipertensão arterial sistêmica (HAS) refratária. A paciente consentiu com a publicação do caso clínico e de suas imagens. O estudo foi aprovado pelo Comitê de Ética local (número do parecer 4.451.756).

## RELATO DE CASO

Mulher de 55 anos, hipertensa, dislipidêmica e portadora de DRC estágio III, em acompanhamento com equipe de cardiologia devido a HAS de difícil controle, em uso de quatro drogas anti-hipertensivas. Foi identificado, durante investigação com Doppler de artérias renais, um volumoso aneurisma em hilo renal direito. Realizou-se ATC de abdome, que demonstrou um AAR tipo II de 3,4 x 2,8 cm, envolvendo a bifurcação da artéria renal principal, que irrigava os polos superior e inferior e um volumoso ramo que nutria o polo médio renal com saída posterior do aneurisma (Figura 1). A paciente apresentava *clearance* de creatinina de 41 mL/min/1,73 m^2^.

**Figura 1:** (A) Angiotomografia com reconstrução volumétrica de aneurisma renal tipo II visão anteroposterior, demonstrando aneurisma renal tipo II acometendo a bifurcação renal; (B) Angiotomografia com reconstrução volumétrica de aneurisma renal tipo II visão posteroanterior, demonstrando aneurisma renal tipo II com ramo nutridor no polo renal médio saindo do aneurisma renal tipo II; (C) Corte angiotomográfico multiplanar demonstrando em 1 – artéria renal principal próximo a bifurcação superior e inferior; em 2 – artéria nutridora do polo médio saindo do aneurisma; (D) Corte angiotomográfico multiplanar demonstrando em 1s – ramo nutridor do polo renal superior; AR – artéria renal principal; (E) Corte angiotomográfico multiplanar demonstrando em 1s – ramo nutridor do polo renal superior; 1i – ramo nutridor do polo renal inferior; 2 – ramo nutridor do polo renal médio.

Após criterioso estudo angiotomográfico renal e estudo das possibilidades de tratamento para maior preservação do parênquima, optou-se pela realização de tratamento endovascular utilizando a técnica de remodelamento com stents em T (t-stenting) e molas para preservação das artérias renais nutridoras. O tratamento foi realizado utilizando introdutor longo 6 Fr Destination (Terumo Medical, Somerset, EUA) com cateterização da artéria renal direita com uso de cateter cobra 2 e fio guia hidrofílico 0,035 x 260 cm (Terumo Medical, Somerset, EUA). Após arteriografia renal com melhor visualização da bifurcação da artéria renal principal (Figura 2A), foi cateterizado o ramo superior da artéria renal principal com uso de fio guia 0,035 x 260 amplatz (Boston Scientific, Minneapolis, EUA), sendo implantado o stent Palmaz Genesis Pro 5 x 39 mm (Cordis Corporation, Warren, EUA) (Figura 2B) justa bifurcação. Na sequência, foi cateterizada a malha do stent Palmaz Genesis Pro (Cordis Corporation, Warren, EUA) com uso de fio guia Pt2 0,014 x 180 cm moderado suporte (Boston Scientific, Minneapolis, EUA) e realizada a cateterização do ramo nutridor do polo médio, sendo implantado um stent Palmaz Blue 3 x 18 mm (Cordis Corporation, Warren, EUA), a partir da malha do stent Genesis Pro para a artéria nutridora do polo médio. Foi realizada, então, cateterização utilizando microcateter Progreat 2.4F (Terumo Medical, Somerset, EUA), entre as malhas do stent, e realizada a liberação de 6 micromolas hidrocoloidais AZUR-18 x 12mm x 20 cm destacáveis (Terumo Medical, Somerset, EUA) sobre o saco aneurismático (Figura 2C). A arteriografia de controle demonstrou preservação dos ramos nutridores renais e embolização do saco aneurismático renal (Figura 2D).

**Figura 2:** (A) Arteriografia renal demonstrando aneurisma renal tipo II complexo – pré-tratamento; (B) Posicionamento do 1º stent justa bifurcação da artéria renal principal; (C) Posicionameto dos stents em T e inicio da embolização vias malhas do stent; (D) Arteriografia de controle final demonstrando ramos nutridores pérvios e embolização do aneurisma renal.

A paciente evoluiu satisfatoriamente no pós-operatório, tendo alta no 3º dia, sem elevação das escorias nitrogenadas ou hematúria, em uso de dupla antiagregação. Realizou ATC no acompanhamento ambulatorial de 3 meses após o procedimento, demonstrando parênquima renal sem isquemia e com todos os ramos nutridores preservados (Figura 3A e 3B). Atualmente, encontra-se em acompanhamento há 3 anos com boa evolução clinica, laboratorial e de imagem. Durante esse período de acompanhamento, não houve a necessidade de terapia substutiva renal, assim como a paciente permaneceu em uso de quatro classes de drogas anti-hipertensivas, mantendo controle pressórico adequado.

**Figura 3:** (A) Controle angiotomográfico com 3 meses demonstrando perviedade do ramo nutridor do polo médio; (B) Controle angiotomográfico com 3 meses demonstrando perviedade do ramo nutridor do polo superior e inferior.

## DISCUSSÃO

Os aneurismas de artérias viscerais (AAV) são raros, presentes em menos de 1% da população total. O AAR é um dos tipos menos frequentes de AAV, correspondendo a cerca de 15 a 22%^4^. Estudos realizados através de autópsia indicam uma incidência de AAR de 0,1%. No entanto, os estudos realizados através da arteriografia renal evidenciam uma incidência que varia de 0,3 a 1%^1^^,^^2^^,^^5^. Essa doença é mais comum no sexo feminino, devido à forte associação com a fibrodisplasia muscular renal^1^^,^^6^, porém também está relacionada a aterosclerose, infecções, trauma, doença de Kawasaki, síndrome de Marfan, displasias vasculares e poliarterite nodosa (PAN)^1^^,^^3^. A maioria dos pacientes é assintomática, mas alguns podem apresentar sintomas como HAS, isquemia renal, hematúria ou dor em flanco, mas a relação é pouco estabelecida. As complicações envolvem hipertensão renal vascular, trombose e embolia renal, infarto renal e ruptura^7^. A ruptura, apesar de um fenômeno relativamente pouco frequente (5 a 10%), associa-se a altas taxas de mortalidade (80%)^1^. Em nosso caso, a descoberta do AAR foi incidental e motivada pela a investigação da HAS refratária de etiologia renovascular.

Trabalhos como os de Barros et al.^1^ e Abath et al.^7^ demonstram que os AAR tipo II são os mais comuns quando comparados com os demais tipos. Esse tipo de aneurisma é definido pela literatura como uma lesão desafiadora, principalmente quando adjacente a bifurcações arteriais, tendo o tratamento cirúrgico aberto (endoaneurismorrafia, nefrectomia ou autotransplante) como opção de tratamento já estabelecido há décadas^6^^,^^7^. Estudos mais recentes recomendam o reparo endovascular através do uso das técnicas de remodelamento com uso de stent associado ao uso de molas ou de molas e agentes embolizantes líquidos, com o objetivo de preservar os vasos renais nativos quando situados em artéria renal principal ou envolvendo bifurcações nos AAR saculares de colo largo^1^^,^^2^^,^^7^. Ainda assim, essas lesões são um desafio para esse tipo de reparo devido à sua difícil anatomia, exigindo técnicas mais complexas^1^^,^^7^. Da mesma forma, os AAR tipo II fusiformes apresentam grande dificuldade de tratamento percutâneo atualmente^1^^,^^7^. Apesar disso, o reparo endovascular tem eficácia comprovada em 89,7 a 98% dos casos, proporcionando uma redução da morbidade, do tempo de operação e do tempo de internação pós-operatória, além de menor trauma cirúrgico^1^^,^^4^^,^^7^.

As indicações para o tratamento do AAR permanecem ainda incertas na literatura, porém há consenso de que existe indicação de tratamento em pacientes sintomáticos, em aneurismas volumosos, com embolizações renais, aneurisma em mulheres grávidas ou em idade fértil e aneurismas > 2,5 cm^1^^,^^2^^,^^6^^-^12.

Até o desenvolvimento das técnicas de remodelamento com stent, os aneurismas saculares de colo largo (proporção saco aneurismático:colo < 2) não podiam ser tratados por embolização seletiva, tendo um risco significativo de oclusão do vaso principal causada pela migração da mola ou do agente embolizante líquido para a artéria desprotegida. Com o surgimento desta técnica, um stent é implantado na artéria principal no colo do aneurisma, enquanto as micromolas ou outros agentes embolizantes são depositados no aneurisma através da malha do stent, que tem um papel de suporte, evitando, assim, a migração inadvertida dos materiais emboligênicos, além de contribuir para alterar os parâmetros hemodinâmicos localmente, redirecionando o fluxo e fornecendo um substrato para endotelização naquela área, o que diminui a chance de recanalização de aneurismas a longo prazo^1^^,^^2^^,^^7^^-^12. Os aneurismas saculares com colo estreito (proporção saco aneurismático:colo > 2) são candidatos para embolização primária do saco aneurismático com molas ou agentes embólicos líquidos^1^^,^^9^^-^12.

A utilização de stents, empregados primariamente em procedimentos de neurointervenção, como o Solitaire (Medtronic, Minneapolis, EUA), apresenta grande navegabilidade e flexibilidade, passando no interior de microcateteres. Inclusive, o stent apresenta a vantagem de poder ser reposicionado mesmo após ter sido completamente liberado. Porém, mais estudos com resultados a longo prazo da utilização desses stents em AAR são necessários^10^. Em nosso caso, não havia a disponibilidade para o uso desses stents.

Os avanços tecnológicos recentes envolvem técnicas endovasculares com stents moduladores de fluxo. Esses stents apresentam múltiplas camadas especificamente desenhadas para reduzir a velocidade de fluxo no interior do saco aneurismático, promovendo trombose e manutenção do fluxo na artéria principal e seus ramos. Eles são amplamente utilizados na neurointervenção, mas sua utilização em vasos periféricos ainda carece de evidências, com pequenas séries e relatos^9^^,^^10^.

No caso relatado, observamos um grande ramo nutridor do polo médio saindo do AAR tipo II, em uma paciente com DRC estagio III, havendo a necessidade de preservação do parênquima renal. Após a análise pormenorizada da ATC em diversas reconstruções e cálculos das artérias aferentes e eferentes do aneurisma, optamos por realizar a preservação da artéria renal utilizando stents de nitinol balão expansíveis, utilizando as malhas do stent como suporte para a preservação do ramo nutridor do polo médio e como apoio para as molas, evitando a migração delas. Após o implante dos stents, preenchemos o saco aneurismático com molas hidrocoloidais no intuito de embolizar o saco aneurismático. O controle arteriográfico intraoperatório e o controle angiotomográfico 3 meses após procedimento demonstraram perviedade dos ramos tratados, ausência de isquemia renal e embolização do saco aneurismático.

A combinação de técnicas que foi realizada para essa paciente é de complexa execução, necessitando de um estudo angiotomográfico rigoroso prévio para avaliação da anatomia e o adequado planejamento do procedimento. Dessa forma, quando a anatomia vascular permitir, é possível reparar AAR tipo II complexo preservando os vasos nativos da topografia renal, como fora realizado neste caso. Não há dúvida de que a presença de uma função renal limítrofe associada a um AAR complexo imponha a necessidade de preservação renal, sendo essa técnica de remodelamento com stents em T e molas bem indicada nesses casos. Atualmente os conhecimentos relacionados a novos materiais e técnicas derivados da neurointervenção tornam o tratamento dos AAR complexos exequível e efetivo, devendo influenciar na escolha terapêutica.
